# *In vivo* quantification of cochlin in glaucomatous DBA/2J mice using optical coherence tomography

**DOI:** 10.1038/srep11092

**Published:** 2015-06-05

**Authors:** Jianhua Wang, Ayman Aljohani, Teresia Carreon, Giovanni Gregori, Sanjoy K. Bhattacharya

**Affiliations:** 1 Bascom Palmer Eye Institute, University of Miami, Miami, Florida, 33136.

## Abstract

The expression of cochlin in the trabecular meshwork (TM) precedes the clinical glaucoma symptoms in DBA/2J mice. The ability to quantify cochlin in the local tissue (TM) offers potential diagnostic and prognostic values. We present two (spectroscopic and magnetomotive) optical coherence tomography (OCT) approaches for *in vivo* cochlin quantification in a periodic manner. The cochlin-antibody OCT signal remains stable for up to 24 hours as seen at 3.5 hours after injection allowing for repeated quantification in the living mouse eyes.

The ability to detect and quantify proteins that are predictors of susceptibility (and/or progression or efficacy of treatments)^1^ in specific local tissue prior to clinical detection will be immensely helpful to control various disease states. The follow up value for such proteins in late onset, progressive, multifactorial or metastatic diseases is immense. The diagnosis using blood or excretory fluids based detection of such proteins is often proven unsuitable due to release of the protein in fluids by multiple organs. Only the local tissue protein content often serves as a true predictor of the given disease or disorder^1^,^2^. Glaucoma refers to a group of late onset, progressive and irreversible blinding diseases where loss of sight occurs without any other previous symptom or pain. In most individuals a significant degree of peripheral vision is lost before the loss is realized. Intervention usually delays its further progression. Glaucoma is frequently associated with elevation in intraocular pressure (IOP). IOP is the only modifiable factor that confers neuroprotection against glaucomatous vision loss even in the glaucoma patients where the IOP is within the normal pressure range (termed normal tension glaucoma)^3^. The trabecular meshwork (TM) is a tiny region in the anterior chamber that undergoes pathologic changes contributing to impeded aqueous humor outflow and consequent IOP elevation.

Mass spectrometric analyses found cochlin in the TM of individuals with glaucoma but not in normal controls^4^. This was also observed in a mouse model of glaucoma (DBA/2J mice), where the elevation of IOP is spontaneous^5^. A mouse with near identical genetic background, DBA/2-Gpnmb+-Sj/J lacks the presence of cochlin in the TM, spontaneous IOP elevation, and glaucomatous neuropathy^6^. A low level of cochlin was detected in the DBA/2J mice preceding IOP elevation^5^. We present strategies for *in vivo* detection of cochlin in the DBA/2J mice using a customized optical coherence tomography (OCT) instrument together with the use of modified cochlin antibodies. The customization combined spectroscopic (SOCT)^7^ and magnetomotive (MMOCT)^8^ imaging approaches in a single instrument. We evaluated proof of principle procedures for OCT quantification of cochlin *in vitro* using polymeric spheres (brain balls; www.marblesthebrainstore) that were subsequently utilized *in vivo* in the eyes of living mouse.

## Results

### Performance of customized optical coherence tomography (OCT) instrument

**Figure 1:**
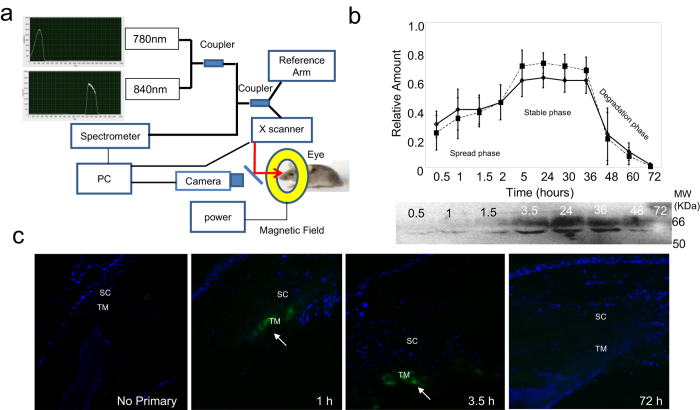
Customized optical coherence tomography (OCT) instrument and optimal imaging time span. (**a**) Schematic diagram of a custom made instrument enabling spectroscopic (with duel light beams at 780 and 840 nm) and magnetomotive imaging. (**b**) Relative amount (signal) determination using NIR dye (solid line; diamonds) and anti-cochlin couple magnetic beads (dashed line; solid squares). The spread, stable and degradation phase in the time span (in hours post-injection) has been shown. Off-line Western analyses at each point has been shown below for indicated time interval (in hours). (**c**) Representative immunohistochemical analyses (20X magnification) of anti-cochlin antibody (detecting cochlin-chicken polyclonal antibody complex in the Trabecular meshwork region in DBA/2J mice. SC = Schlemm’s canal; TM = Trabecular meshwork. Immunoreactivity has been shown by an arrow. The time in hours indicates post-injection time. A no primary antibody has been shown as a control.

The schematic diagram of our OCT device is presented in Fig. 1a. The spectroscopic OCT harbors two discreet light sources at 780 and 840 nm with the bandwidth of ±10 nm (Fig. 1a, Supplementary Fig. 1a). We evaluated the difference in SOCT image using these two wavelengths using a droplet of water and a droplet of infrared (IR-780 nm) dye coupled-antibody. Water shows a similar image at both wavelengths (Supplementary Fig. 1b) but the image with IR780 dye shows a markedly lower OCT signal in the OCT at 780 nm (Supplementary Fig. 1b). The 840 nm SOCT image serves as control. Within a polymeric sphere, the image with IR780 nm dye subtracted from that without the dye correlates with the magnitude of absorbance due to the dye. In polymeric spheres or in eyes the IR dye will correlate with antigen-antibody complex. It is possible to determine the magnitude of signal absorbed, which correlates with the amount of antigen-antibody complex. A series of two-dimensional images enables averaging and quantification of the absorbed signal. The absorbance normalized for slight variation in the area, provides a quantitative relationship with the amount of dye alone in a polymeric sphere (Supplementary Fig. 1c) and the same is expected for dye coupled-antibody.

The antibody (anti-cochlin) coupled magnetic nanoparticles that forms a complex with the antigen (cochlin) undergoes a change in orientation under the influence of a magnetic field (Fig. 1a), which results in changes in the scattering properties around the affected molecules (Supplementary Fig. 1d). The magnetic bead coupled antibody-antigen complex registers a distinctly different scattering in the magnetic “off” position compared to the “on” position. This was evaluated *in vitro* using polymeric spheres with or without injection of the antigen-antibody complex (Supplementary Fig. 1e). The difference between the “off” and “on” images reflects the presence of the magnetic bead coupled antibody-antigen complex. Our *in vitro* evaluations utilized polymeric spheres that expand depending upon the time interval for water soaking (Supplementary Fig. 1f). The un-soaked spheres are smaller and attain the size of a mouse eye after ~45 seconds of soaking in the water (Supplementary Fig. 1f). These spheres were used for MMOCT imaging with different amounts of cochlin-antibody complex (Supplementary Fig. 1g). Injected sequentially (antigen then antibody) or in a premixed manner, a linear response curve can be derived from MMOCT images with increasing amounts of cochlin (Supplementary Fig. 1h).

We determined the optimal time for periodic *in vivo* cochlin quantification. Injections of IR780-coupled (Supplementary Fig. 2a, b) or magnetic nanoparticles coupled antibodies (Supplementary Fig. 2c, d) frequently result in a focal edema immediately after injection (Supplementary Fig 2a, b, blue images). The distortion of after injection compared to control before injection images may cause a mismatch in image superimposition (Supplementary Fig. 2a, b, overlay of blue and red images). An incubation period post-injection leads to spreading of antibodies, induces a “wash out” effect, substantially reduces edema, and enables better image superimposition. We use the term “wash out” to indicate that antigen-bound antibody remains while free antibody is either substantially diluted or removed from the local tissue resulting in providing a more specific signal. Images that were taken from ~20 μm from the site of injection provided good quality after injection images (similar to raster scan; 20 OCT-B scan per image) for improved superimposition. Images were acquired for all across 360° except for a region of about ~20 μm around the injection site. We found injecting the antibody at every 90° rotating along the optical axis of the eye is optimal for obtaining quantifiable images irrespective of mouse age, gender and state of IOP. A slight variation in the antibody spread (IR or magnetic bead coupled) is found from mouse to mouse. The aqueous humor outflow has been found to be segmental^9^, which is consistent with our initial observation of cochlin deposits^4^. We found an incubation of ~3.5 hours achieves uniform antibody spreading (irrespective of IR-780 or nanoparticle coupled) with every 90° injection (4 injections total) for DBA/2J mice with a range of cochlin in their TM (Fig. 1b). The cochlin-antibody complex signal usually remained stable for over 24 hours (Fig. 1b). In a limited subset of mice (less than 1%; n = 100), the SOCT signal decreased slightly more than 15% of the initial signal (at 3.5 hours) between approximately 16–24 hours after injection. The signal starts fading rapidly after 30–36 hours and by 72 hours we find a complete loss of signal in all tested animals (Fig. 1b). These results were confirmed using endpoint offline Western blot and immunohistochemical analyses for antibody degradation (Fig. 1b,c).

### Spectroscopic OCT imaging and quantification of cochlin at different ages as well as different intraocular pressure (IOP) in DBA/2J mice eyes

**Figure 2:**
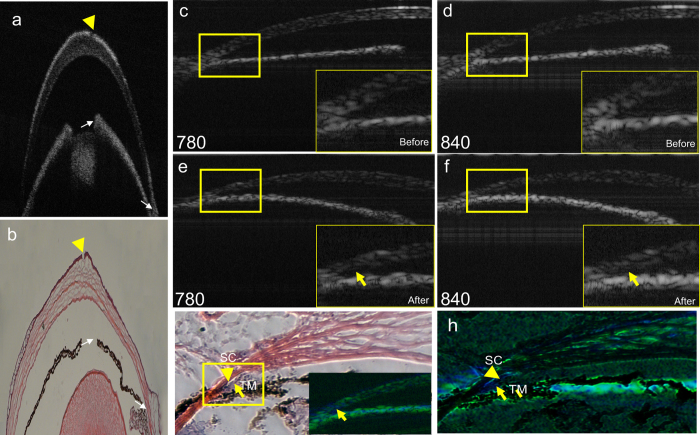
Spectroscopic OCT imaging of DBA-2J mice eyes. Infrared (IR) dye coupled anti-cochlin injection was performed for these imaging. (**a,b**) The natural landmarks (iris boundary and iridocorneal angle) in OCT and anatomic images indicated by white arrows. IR dye injection with a blunt needle and the corresponding dent (arrowhead) has been shown in OCT (a) and in anatomic image (b) respectively. (**c–f**) Eyes was imaged before and after injection with anti-cochlin-IR 780 nm dye (indicated) with 780 or 840 nm SOCT light source as indicated, arrow show light absorption. (**g**) Anatomic image [Hematoxylin-eosin (H&E) stain], arrow and arrowhead shows TM and SC. *In situ*: superimposed image of 780 (blue; before) and 840 (green; after IR dye injection). (**h**) Superimposed SOCT and anatomic image with TM region (arrow; blue) after injection, arrowhead indicate SC region.

**Figure 3:**
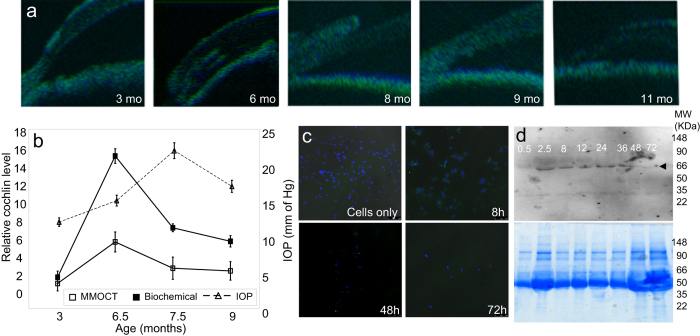
Quantification of cochlin in DBA/2J mice at different ages and with different intraocular pressure (IOP). (**a**) Representative MMOCT images (Off and On superimposed) DBA/2J mice at indicated ages (**b**) Representative estimation of cochlin in mice at 3–9 months of age with indicated IOP using MMOCT approach and endpoint biochemical (Western blot) analyses. Mean± standard deviation from n = 10 animals. (**c**) Immunohistochemical analyses for *in vitro* anti-cochlin IgY-cochlin complex clearance in cell culture after exogenous recombinant cochlin-anti-cochlin addition. (**d**) Western blot analyses of the media for determination of clearance of cochlin-anti-cochlin IgY complex *in vitro* in cell culture. Arrow indicates position of anti-IgY. Bottom panel shows an identical Coommassie blue stained gel.

In an effort to quantify total cochlin localized in the TM, we preferred to match the stacks of images (full OCT datasets) before and after antibody injections for both SOCT and MMOCT modalities. We utilized natural landmarks, such as the boundary of the iris and iridocorneal junction (Fig. 2a, white arrows). In addition, we used concentrated IR780 dye that helps create an absorbed spot with lack of signal (Fig. 2a, arrowhead). Such injection is flexible and operator defined. Using a blunt needle, while injecting the IR dye, we created a dent, which was evident in the anatomic images (Fig. 2b, arrowhead). The SOCT image was acquired before and after injection of the 780 nm IR-dye (Fig. 2c–f) using the 780 nm (Fig. 2c,e) as well as 840 nm light source (Fig. 2d,f). The image with the 780 nm light source shows a difference at the iridocorneal joint region (Fig.2e *in-situ* indicated by arrow). The superimposition of the image prior to injection (blue) on the image after injection (green) shows appearance of blue in the TM region in the superimposed image (Fig. 2g, inset, arrow). In order to determine whether this bluish region in the iredo-corneal angle was indeed the TM region, we superimposed the OCT image and compared it with the anatomic image (Fig. 2g; boxed bluish region, arrow; Schlemm’s canal is indicated by arrowhead). A superimposition of two OCT images (before and after injection; blue and green respectively) with the same size anatomic image [hematoxylin-eosin (H&E) stained as in Fig. 2g] presents a dark blue color in the TM region (arrow) confirming detection of cochlin in the mouse TM with OCT (Fig. 2h). This superimposition was achieved using natural landmarks (Fig. 2a,b). In corresponding anatomic images (Fig. 2b,g) additional landmarks corresponding to IR dye can be achieved by co-injecting trypan blue/bromophenol blue and UV cross-linking, which helps induce dye retention during processing. Unique anatomic features detectable in OCT images can also be used as landmarks. The two natural landmarks (boundaries; Fig. 2a,b white arrows) combined with a third landmark (Fig. 2a,b arrowheads) are generally sufficient for alignment. These matches showed the OCT bluish region aligned with the bluish region of the hematoxylin-eosin (H&E) image corresponding to trabecular meshwork in mice (Fig. 2h) corroborating the presence of cochlin in our previous studies^6^. OCT images taken at every 3° throughout the full 360° proved to be the most optimal procedure for cochlin quantification compared to other images that were taken (data not shown). Custom software was developed in Matlab to register and compare images (Supplementary Fig. 2e). We used en face OCT images to register the “On” and “Off” 3D OCT datasets at a fixed time point. We used anatomical features visible on these enface images to manually register the OCT datasets at different time points and derive the relevant qualitative results (Supplementary Fig. 2f,g). We also compared them to images obtained with a microscope (Phoenix Research Laboratories, Pleasanton, CA) specifically designed to image the TM region (Fig. 2h) to identify the location of the TM in the OCT scans. We then determined normalized OCT signal intensity for cochlin in the appropriate regions. We imaged mice of various ages using these OCT methods (Fig. 3a). We carefully selected mice (at specific age with very close IOP range) at age 3–9 months grouped in ages 3, 6.5, 7.5 and 9 month intervals that had elevated IOP around 7.5 months and slight decline from peak elevated IOP at 9 months. All of these mice also had none to very little pigmentary dispersion as determined by non-invasive TM microscopic imaging and an open anterior chamber angle determined using OCT. For initial assessment, we located iridocorneal angle and used approximately 250 μm in each direction (sclera and cornea side) for segmentation and cochlin quantification. This segmentation region may well include sclera or cornea but since cochlin is not present in either tissue it gives a good approximation. We took a second approach that is locating lumen and boundaries of Schlemm’s canal (SC) and then encompassing 200 μm from the boundary of SC on either side. The measurements utilizing this approach were used for actual quantification. Regions of interests were manually delineated on the registered datasets to derive the relevant quantitative results (Supplementary Fig. 2f, g). We found peak cochlin amount occurs just before the peak IOP in these mice determined using MMOCT imaging *in vivo* and endpoint (mouse were euthanized after MMOCT imaging) single biochemical measurements *in vitro* (Fig. 3b). The MMOCT measurement trends are in agreement with offline biochemical measurements (Fig. 3b).

We observed stable cochlin-antibody complex in the TM from 3.5–30 hours and its subsequent degradation (Fig. 1b). We sought to determine if the degradation kinetics is parallel *in vitro* and whether it occurs predominantly at the extracellular matrix (ECM), the site of cochlin secretion. Immunohistochemical (Fig. 3c) and Western blot analyses (Fig. 3d) using exogenous recombinant cochlin-antibody and primary TM cell cultures suggests slightly more rapid degradation of the antibody *in vitro* after 24 hours otherwise the former parallels the *in vivo* degradation time course (Figs. 1b and 3d). Our results are consistent with degradation of the cochlin-antibody complex predominantly in the ECM (Fig. 3c) Our results suggests the periodicity of the cochlin measurement will need at least a 72 hours gap period between first determination and injection for subsequent quantification.

## Discussion

High throughput and in particular, OMICS approaches have now opened up the possibility to identify proteins or other biomolecules that may serve as predictors of susceptibility, progression and efficacy of treatment^1^. Periodic quantification of such protein predictors in late onset and progressive diseases such as glaucoma is critical for intervention in these diseases. A number of imaging modalities have emerged enabling detection of biomolecules in the tissues and rendition of 3D images. However, the choice of detection and quantification of proteins in the local tissue *in vivo* (in living organisms) is rather limited to a handful of methods such as positron emission tomography (PET) or nuclear magnetic resonance (NMR)^10^,^11^,^12^,^13^,^14^. The anterior eye segment is a spatially accessible tissue and enables imaging even with low penetrating power. The approach presented here is minimally invasive and, as proof of principle, we show it enables repeated imaging to monitor the protein cochlin. This modified imaging method could be easily extended to other superficial tissues such as other areas of eye, skin, mouth cavity etc. For imaging in humans longer wavelengths such as 1200–1500 nm range may be necessary. The depth of the anterior segment tissues in human is greater than that in the mouse. Longer wavelengths have deeper tissue penetration^15^ and will be more suitable for use in humans. Such reagents are readily available and the device could be easily tailored to such needs. Our technique could be extended for detection of a variety of biomolecules not only proteins. We have also considered future use of these two modalities for detecting protein-protein or protein-lipid/metabolite interactions. A third modality, plane polarized light can also be used in principle. Several marine shrimps possess chromatic particles that respond to plane polarized light^16^,^17^. The use of the OCT with these modalities could be used for simultaneous detection of at least three different molecules independently and/or confirmation of interactions among them. However, important caveats and limitations remain in OCT based protein quantification approaches. We have used differences in 780 nm images taken before and after IR coupled dye injection (Fig. 2c,e). We have used natural landmarks for comparison of images taken at different time points, a practice adopted from published reports for longitudinal comparison of patient images in clinical settings^18^,^19^,^20^. The caveat of this approach is that since the images are taken at two different times, thus despite the use of landmarks it is very difficult to image the exact same location and some differences in images remain. Another limitation is the small differences in the angle of incidence of scanning beam, which also results in some changes in OCT signal. Variation in light beam absorption with scan depth is another limitation that may still introduce errors in protein concentration estimation across different depths of the tissue. Multiplication of difference images across scan depth with a correction factor derived from estimates of differences in light beam absorption as a function of depth can help eliminate this error. An approach is under development for calibrating intensity drop ratio independent of scan depth, which in future may help rectify this problem. These differences are sources of errors in the estimation of cochlin concentration and remains as limitations of the current approaches.

## Methods

The study protocols were approved by the University of Miami IACUC. The methods were carried out in accordance with the approved guidelines.

### Mouse colonies and general procedures

The DBA/2J and DBA/2-Gpnmb+-Sj/J mouse colonies were maintained and utilized following institutional animal care and use committee approved protocols. Mice were anaesthetized using intraperitoneal injections of ketamine and Xylazine (90 and 10 mg per Kg). The intraocular pressure (IOP) measurements were carried out using TonoLab (Colonial Medical Supplies Co., NH). Selected measurements were confirmed using cannulation method^4^. A phosphate buffered saline (PBS) drop was applied to the eyes prior to IOP measurements.

### Optical coherence tomography and image analyses

The instrument constructed for these studies is a combined Spectroscopic (S) and magnetomotive (MM) OCT. The SOCT instrument described previously^21^,^22^ was modified to incorporate two intense beam of light sources 780 ± 10 nm (SLD 381, Superlumdiodes Ltd, Moscow Russia) with an output power of 0.421 mW and 840 ± 10 nm trimmed from a 100 nm bandwidth light source (Broadlighter, BLM2-D-840, Superlumdiodes Ltd, Moscow Russia) with an output power of 0.928 mW respectively. The MMOCT instrument run with the full bandwidth (100 nm) of the BLM2 light source centered at 840 nm. The IR-780 dye was custom conjugated with chicken polyclonal cochlin antibodies^23^ using services of Rockland Inc., Boyertown, PA. The anti-cochlin chicken polyclonal antibodies were raised using services of Aves Lab Inc., Portland, OR as described previously^23^. The antibodies were re-suspended in PBS as needed. Polymeric expandable spheres (originally procured from www.marblesthebrainstore.com) were a research gift from Dr. Richard K. Lee. These spheres also called as braindrops balloons to 200X of its original size and were used for initial optimization studies instead of mouse eyes (Supplementary Fig. 1f). In polymeric spheres cochlin (0.01–1 μg in 1 μl injection volume) was followed by sequential injection of a slight excess (1.1–1.5 fold) in the same location determined optimal sensitivity range as 0.01 μg to 0.05 μg cochlin (Supplementary Fig 1g and h). The linear range of average signal intensity was determined averaging 20 images.

A powerful magnetic coil (450 Tesla; MC-P88 magnetic coil; Hangzhou Mingzhe Magnetic Tech Co. Ltd, Hangzhou, China, web: http://hzmzcd.cn.china.cn/contact-information/) was added to the instrument. This magnetic coil is more powerful from our previous stand alone MMOCT device^24^. A cooling coil circulating water from a 24 °C water bath maintained the temperature of coil during extended image acquisition. Custom generated chicken polyclonal antibodies to cochlin (Aves Labs, Portland, OR) described previously^23^ were coupled with magnetizable iron nanoparticles of various sizes (5, 50, 100, 150, 250, 500, 800 and 1000 nm average diameter) using custom bioconjugation services of Nanocs Inc., New York, NY. Size of nanoparticles affects OCT signal,150–500 nm is optimal. Below 20 nm and beyond 800 nm results in lack of signal and impaired antibody spreading respectively. The particles larger than 500 nm require longer time for clearance when injected in tissues. The quantification of protein in the TM necessitates 20 cross sectional OCT image with distance between each image 0.005 inch (~.013 μm). Custom software was developed to compute the differential intensity at TM location.

### Anatomic, OCT imaging and image processing

For the anatomic image (Fig. 2b, lower panel), the eye was enucleated immediately after OCT imaging and was embedded in optimal cutting temperature medium without fixation. The eye was snap frozen and sectioned on a microtome. Thus OCT (Fig. 2a) and anatomic images (Fig. 2b) were somewhat different in appearance due to processing induced changes. The injections for anti-cochlin chicken antibodies conjugated with IR 780 or magnetic beads were made using a 36-gauge needle in the TM region by an experienced ophthalmologist. Image acquisition details are described within the manuscript text. Images were analyzed using custom Matlab software.

### Cell culture experiments

Primary TM cells were isolated from 10 Caucasian donor eyes age range 51 ± 7 years of either gender following established protocols^4^. The TM cells in 12 well slide wells were incubated with cochlin-antibody (IR 780 dye or nanoparticle coupled) complex for various time intervals as indicated in specific experimental results and subjected to immunohistochemical analyses using a Leica TSP5 confocal microscope and Alexa fluorophore conjugated anti-chicken antibodies raised in goat. Western analyses of medium from the culture was probed for anti-chicken antibody followed previously published protocols^23^.

### Western blot and Immunohistochemical analyses

Western blot analyses was performed using 10 μg protein, anti-chicken polyclonal antibodies raised in goat coupled with horse radish peroxidase using established protocols^23^. For Immunohistochemistry, the anti-chicken polyclonal antibodies raised in goat were coupled with Alexa594 or Alexa488 following protocols routinely used in our laboratory^23^.

## Additional Information

**How to cite this article**: Wang, J. *et al.* *In vivo* quantification of cochlin in glaucomatous DBA/2J mice using optical coherence tomography. *Sci. Rep.* **5**, 11092; doi: 10.1038/srep11092 (2015).

## Supplementary Material

Supplementary Information
