# Supplementary material for: In vivo quantification of cochlin in glaucomatous DBA/2J mice using optical coherence tomography
Source: Sci Rep. 2015 Jun 5;5:11092. doi: 10.1038/srep11092 (PMC4457137; doi:10.1038/srep11092)
Supplement: Supplementary Information [file srep11092-s1.pdf]

# In vivo quantification of cochlin in glaucomatous DBA/2J mice using optical coherence tomography.

Jianhua Wang, Ayman Aljohani, Teresia Carreon, Giovanni Gregori and Sanjoy K. Bhattacharya*

## Supplementary Information

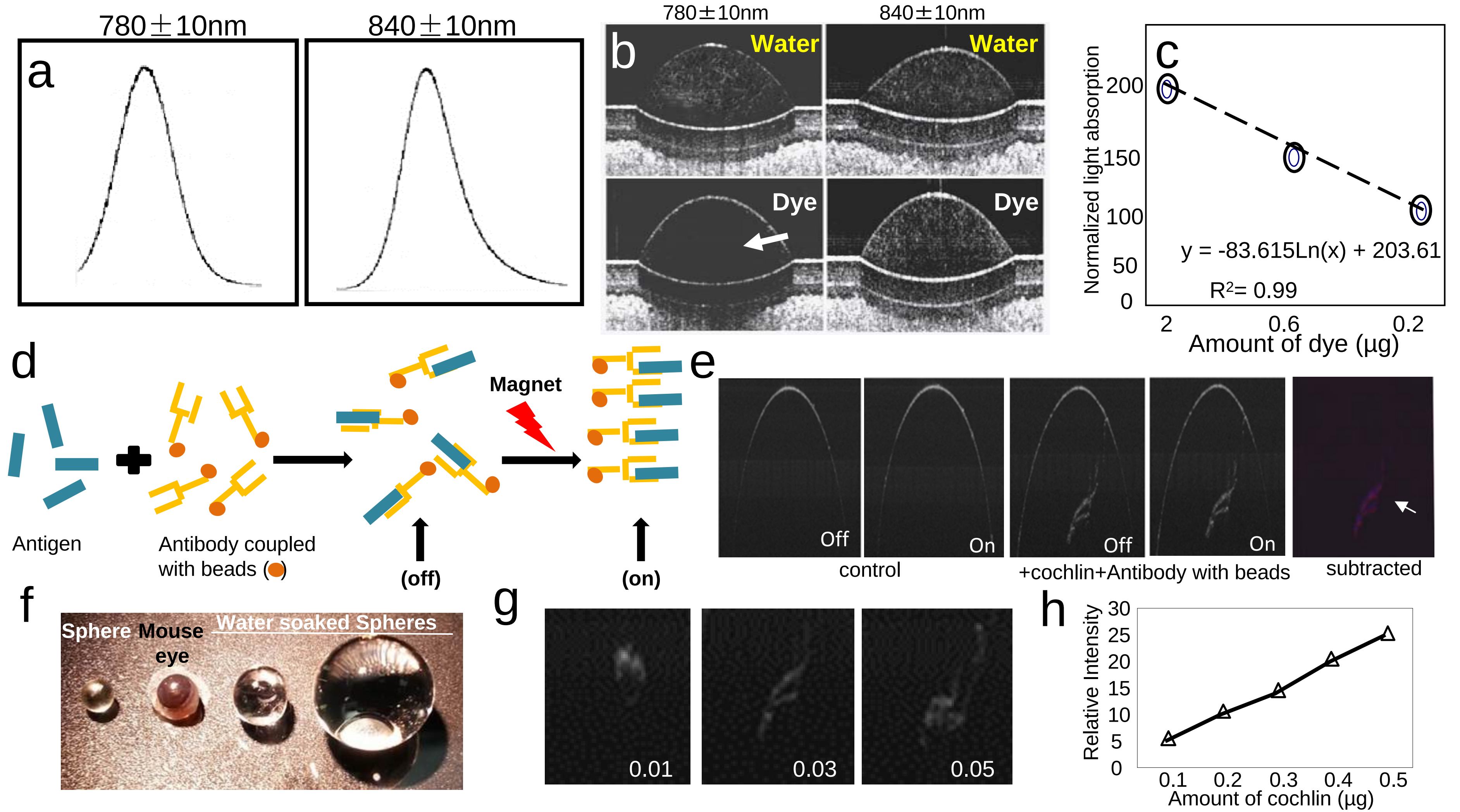


**Supplementary Figure 1** Concept of relative quantification using SOCT and MMOCT. SOCT concepts (**a**) Spectra of the SLD light sources as indicated. 780nm corresponds to the NIR dye absorption wavelength. (**b**) Representative image of a drop of water or NIR dye using light sources as indicated. Dye shows relative lack of scatter at 780nm. (**c**) Relative quantification of light absorption (arbitrary units) as a function of different dye concentrations within polymeric spheres. MMOCT concepts (**d**) Schematic diagram to show the orientation changes of antibody-magnetic beads due to magnetic field (off and on results in changes in OCT signal). (**e**) Representative images of control and injected [cochlin (0.03µg)+antibody coupled magnetic beads (1µl)] polymeric spheres with off and on magnetic field as indicated with a subtracted (Off-On) image for injected sphere. (**f**) Representative image of polymeric spheres with different degree of water soaking and a mouse eye. (**g**) Representative subtracted (Off-On) image of spheres injected with cochlin (0.01- 0.05 µg as indicated) +anti-cochlin coupled magnetic beads. (**h**) Relative intensity determined from Off-On MMOCT images as in G for different cochlin amounts.

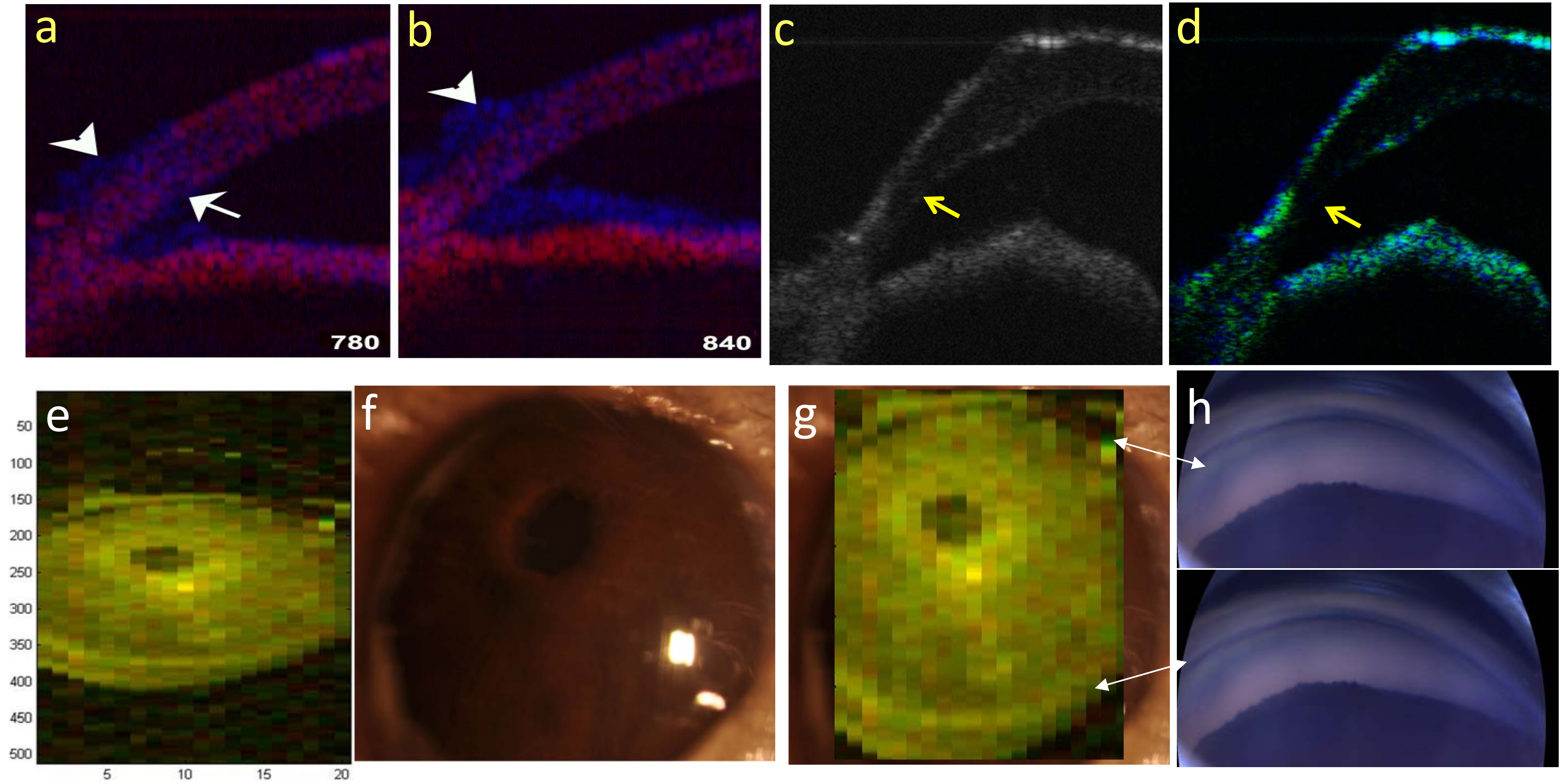


**Supplementary Figure 2** Image superimposition strategies. SOCT images of mouse eyes injected with anti-cochlin coupled fluorophore with absorbance at 780 nm. (**a**) OCT was used with a light source for 780±10 nm and (**b**) with an 840±10 nm light source as indicated. Image before (red) and after injection with flurophore (blue), the arrow head shows the edema that results immediately after injection. The arrow shows the TM region where the antibody is concentrated. Post-injection signal difference is prominent at 780 nm but not at 840 nm wavelength. Several transforms such as traphine and quadratic attempted for superimposition. (**c**) Representative MMOCT image of a mouse eyes injected with anti-cochlin coupled with magnetic beads with magnet power on (**d**) Superimposed On (blue) and Off (green), arrow show more signal at TM area with the magnet power "On". (**e**) A software generated OCT image for generation quantification. (**f**) Representative anatomic image for identification of location of regions from OCT images. (**g**) Representative superimposition of anatomic and image generated from OCT image analyses. (**h**) A special microscope generated image of TM region in the anterior chamber for identification of TM region in the software generated 3D OCT image stack.

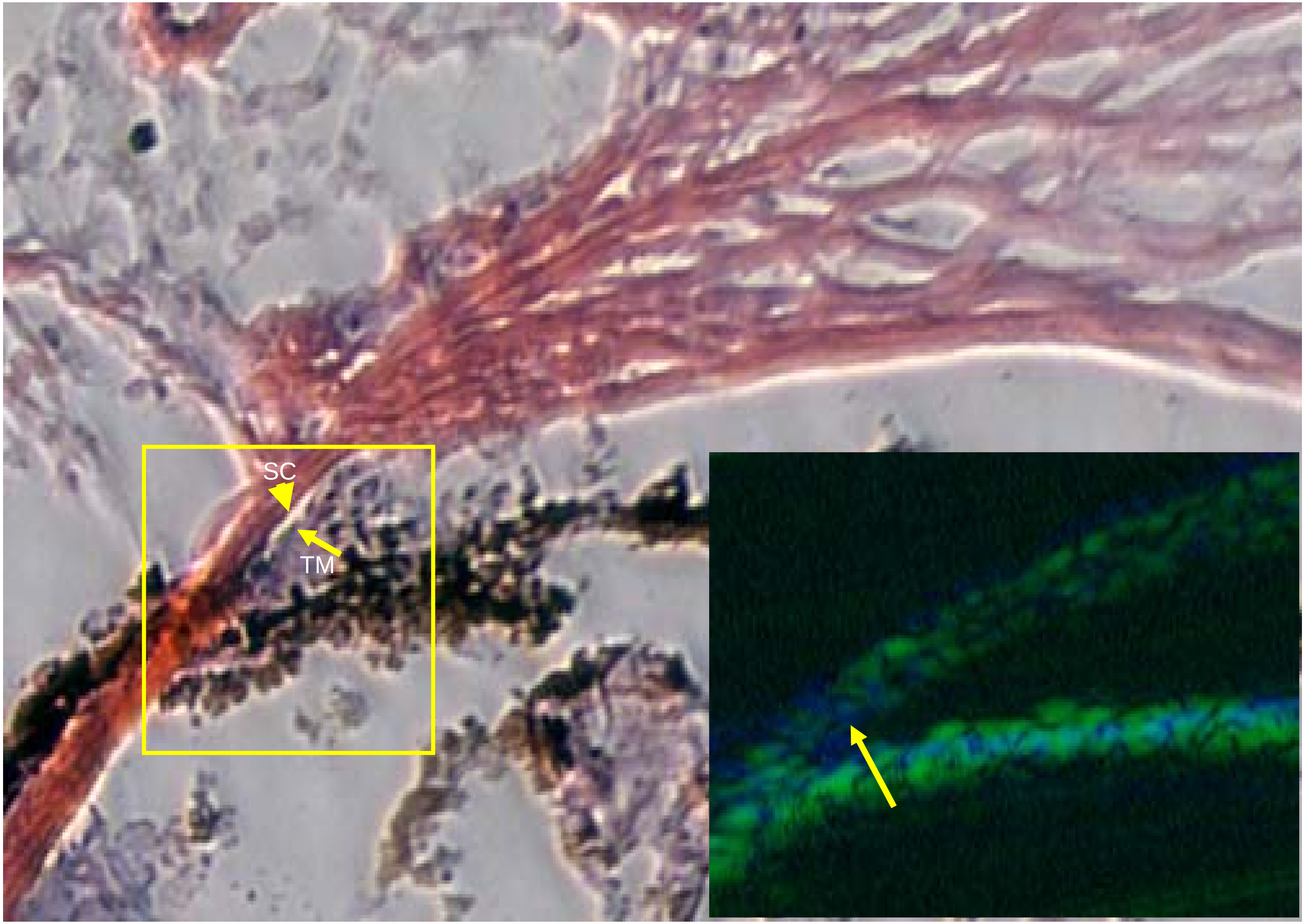


**Supplementary Figure 3 (An enlarged version of Figure 2g)** Anatomic image [Hematoxylin-eosin (H&E) stain], arrow and arrowhead shows TM and SC. In situ: superimposed image of 780 (blue; before) and 840 (green; after IR dye injection). Arrow head and arrow: Schlemm's canal (SC) and Trabecular meshwork (TM) as indicated.

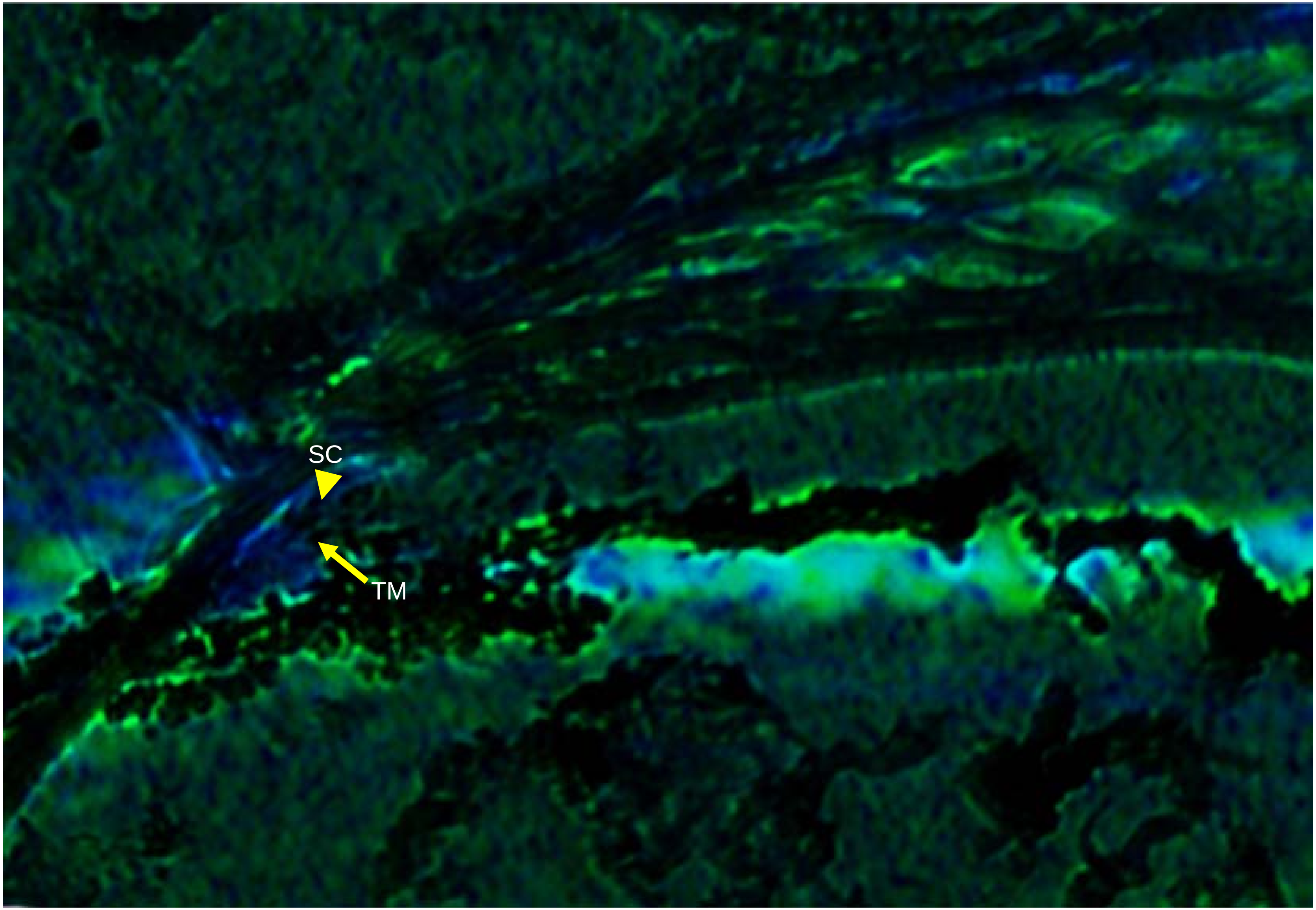


**Supplementary Figure 4 (An enlarged version of Figure 2h)** Superimposed SOCT and anatomic image with Trabecular meshwork ™ region (arrow; blue) after injection, arrowhead indicate Schlemm’s canal (SC) region.

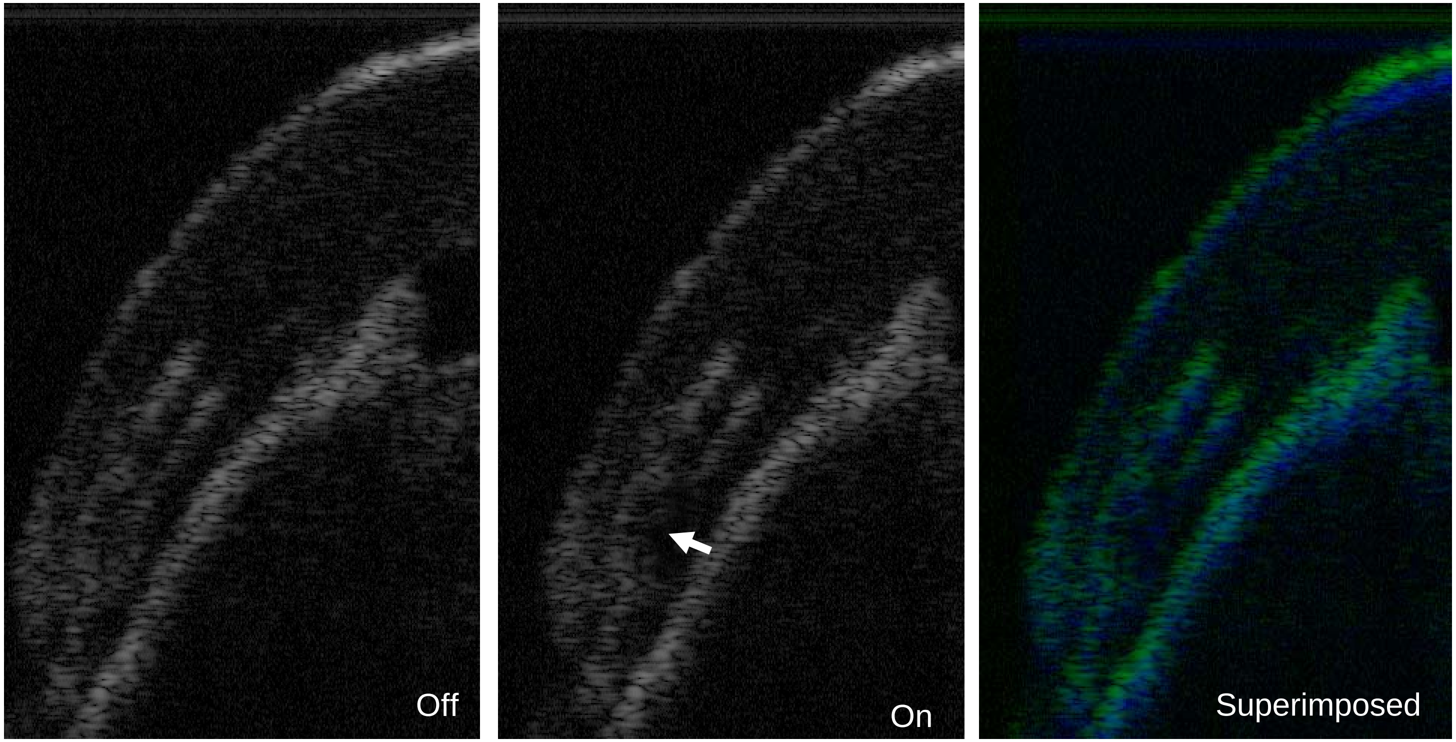


**Supplementary Figure 5** Representative MMOCT images (Off and On superimposed) DBA/2J mice at 6 months of age. The area where the signal undergoes a significant change is indicated by the arrow.
